# Lebenszeitprävalenz des Erlebens von Sex und sexueller Berührung gegen den eigenen Willen sowie Zusammenhänge mit gesundheitsbezogenen Faktoren: Ergebnisse aus der Studie Gesundheit und Sexualität in Deutschland (GeSiD)

**DOI:** 10.1007/s00103-021-03434-6

**Published:** 2021-10-18

**Authors:** Franziska Brunner, Safiye Tozdan, Verena Klein, Arne Dekker, Peer Briken

**Affiliations:** Institut für Sexualforschung, Sexualmedizin & Forensische Psychiatrie, Universitätsklinikum Hamburg-Eppendorf, Martinistraße 52, 20246 Hamburg, Deutschland

**Keywords:** Sexuelle Gewalt, Sexueller Kindesmissbrauch, Lebensqualität, Häufigkeit, Trauma, Sexual violence, Child sexual abuse, Trauma, Quality of life, Frequency

## Abstract

**Hintergrund:**

Die negativen Auswirkungen von sexueller Gewalt auf die Gesundheit sind im vergangenen Jahrzehnt weltweit erneut stark in den gesundheitspolitischen Fokus gerückt. Bislang fehlen für Deutschland bevölkerungsrepräsentative Daten, auf deren Basis die Lebenszeitprävalenz für unterschiedliche Altersgruppen sowie spezifische Zusammenhänge zu gesundheitsbezogenen Faktoren dargestellt werden können.

**Ziel der Arbeit:**

Die Studie untersucht 1) die Lebenszeitprävalenz für Sex sowie sexuelle Berührung gegen den eigenen Willen im Kindesalter und über die Lebensspanne sowie 2) die Zusammenhänge mit gesundheitsbezogenen Faktoren.

**Material und Methoden:**

4955 Personen im Alter von 18 bis 75 Jahren wurden in einer zweistufig geschichteten, randomisierten Einwohnermeldeamtsstichprobe im Rahmen des bundesweiten wissenschaftlichen Survey „Gesundheit und Sexualität in Deutschland“ (GeSiD) befragt. Die Zusammenhänge mit soziodemografischen und gesundheitsbezogenen Faktoren wurden (altersadjustiert und stratifiziert für Geschlecht) mittels logistischer Regression berechnet.

**Ergebnisse:**

Für Frauen lag die Lebenszeitprävalenz für (versuchten/vollzogenen) Sex bei 14,9 % und für (versuchte/vollzogene) sexuelle Berührung gegen den Willen bei 40,8 %, für Männer bei 3,1 % respektive 13,2 %. Für erzwungenen Sex vor dem 14. Lebensjahr lag die Prävalenz bei 2,1 %, für sexuelle Berührung bei 7,5 %. Es zeigten sich höhere Prävalenzen bei Personen mit beeinträchtigter Lebensqualität, schlechtem Gesundheitszustand, chronischer Erkrankung oder Behinderung, einer Behandlung aufgrund von Depression oder einer anderen psychischen Störung im letzten Jahr.

**Diskussion:**

Die Studie verdeutlicht Zusammenhänge von sexueller Gewalt mit psychischer und somatischer Gesundheit. Sie unterstreicht die Dringlichkeit, nach solchen Erfahrungen regelhaft in der ärztlichen Anamnese zu fragen.

**Zusatzmaterial online:**

Zusätzliche Informationen sind in der Online-Version dieses Artikels (10.1007/s00103-021-03434-6) enthalten.

## Abstract (en)

**Background:**

In the last decade, the negative effects of sexual violence on health have again become a focus of health policies worldwide. So far, representative population data on lifetime prevalence for different age groups and specific links to health-related factors for Germany are lacking.

**Objectives:**

The study aimed to assess 1) the lifetime prevalence of self-reported nonconsensual sexual intercourse and sexual touch in childhood and across the lifespan and 2) associations with health-related factors.

**Materials and methods:**

Using a two-step stratified, randomized sample of residents’ registration offices, 4955 persons aged 18 to 75 years were interviewed within the scope of the nationwide scientific survey on health and sexuality in Germany (GeSiD). The associations between experiences of sexual violence and socio-demographic and health-related factors were age-adjusted and stratified for gender using logistic regression.

**Results:**

For women, the lifetime prevalence of (attempted/completed) nonconsensual intercourse was 14.9% and in the form of (attempted/completed) nonconsensual sexual touching was 40.8%; for men the prevalences were 3.1% and 13.2%, respectively. Regarding forced experiences in childhood, the prevalence of sex was 2.1% and of sexual touching was 7.5%. For both genders, the prevalence of nonconsensual intercourse was significantly higher in the case of low quality of life, poor health condition, a chronic illness or disability, or treatment for depression or for another mental disorder.

**Conclusions:**

The results illustrate the strong links between sexual violence and mental and somatic health. This points to the urgency to routinely explore experiences of sexual aggression.

## Einleitung

Die möglichen Folgen sexueller Gewalt umfassen körperliche, sexuelle und psychische Störungen und sind empirisch gut belegt [1–7]. Bei Personen, die traumatische sexuelle Ereignisse bereits im Kindesalter erlebt haben, lassen sich funktionelle und hirnmorphologische Veränderungen nachweisen, welche wiederum mit Störungen im Bereich des emotionalen Erlebens und der Affektregulation in Verbindung gebracht werden (für eine Übersicht s. [8]). Dies kann die Zusammenhänge zwischen sexuellem Kindesmissbrauch und der Entwicklung einer Depression im Erwachsenenalter erklären [9]. Auch für eine Reihe chronischer (psycho)somatischer Erkrankungen wie funktionale gastrointestinale Störungen, unspezifische Schmerzstörungen, chronischen Beckenbodenschmerz, psychogene Anfälle oder Fibromyalgie konnte ein Zusammenhang mit dem Erleben von sexuellem Kindesmissbrauch nachgewiesen werden (für eine Übersicht s. [10]). Die schädlichen Auswirkungen auf die psychische und somatische Gesundheit sind jedoch keinesfalls allein auf sexuelle Gewalterlebnisse im Kindesalter beschränkt, es lassen sich diese auch bei später im Leben stattfindenden sexuellen Gewalterfahrungen nachweisen [1–5, 11–14].

Frauen sind weltweit einem höheren Risiko ausgesetzt, sexuelle Gewalt zu erfahren, als Männer [1]. Daher erscheint auch die Studienlage für Frauen deutlich besser als für Männer [7, 15, 16]. Studien mit Blick auf Geschlechterunterschiede weisen auf Unterschiede in den gesundheitlichen Auswirkungen hin [7, 11, 12]. Insgesamt legen die bisher genannten Befunde einerseits nahe, dass die Beeinträchtigung der Gesundheit die Folge sexueller Gewalt bei beiden Geschlechtern sein kann, wobei eine Studie einen Zusammenhang mit einem selbstberichteten schlechten Gesundheitszustand nur für Frauen und nicht für Männer findet [17]. Menschen können auch infolge von Krankheit oder Behinderung ein erhöhtes Risiko haben, sexuelle Gewalt zu erleben. Fegert und Kolleg:innen nennen als Beispiele Fälle von sexuellen Übergriffen auf Kinder, Jugendliche und Erwachsene in der Psychiatrie, der Psychotherapie, der Altenpflege, bei körperlicher Diagnostik oder während der Krankenbehandlung unter Narkose [18].

Die Erfassung der bundesweiten Prävalenz sexueller Gewalt hat sowohl hinsichtlich eines verbesserten Angebots und Schutzes bereits geschädigter Personen als auch zur Vorbeugung zukünftiger Gewalttaten große Bedeutung. Zuverlässige Häufigkeitsangaben können beispielsweise zur Identifikation von Risikofaktoren oder zum Monitoring von Häufigkeitsveränderungen über bestimmte Zeiträume genutzt werden. Sie tragen somit zu einem verbesserten Verständnis der Entstehung und der Kontextfaktoren von sexueller Gewalt bei. Für die Planung von spezifischen Behandlungs- und Präventionsprogrammen ist es wichtig, die Häufigkeit von sexueller Gewalt nicht nur für die Allgemeinbevölkerung, sondern auch in spezifischen Gruppen (z. B. unterschiedliche Alterskohorten, Geschlechter, psychisch und somatisch Erkrankte) abschätzen zu können. Für Deutschland liegen derzeit nur eingeschränkt aussagekräftige Prävalenzangaben für sexuelle Gewalterfahrungen vor. Einerseits ist die Anzahl bevölkerungsrepräsentativer Studien sehr überschaubar, andererseits variiert darin die Form der Erfassung von sexueller Gewalt bzw. sexueller Grenzverletzung.

Für die Häufigkeitsangaben zu sexuellem Kindesmissbrauch wurde die bisherige Studienlage in einer Expertise im Auftrag des Unabhängigen Beauftragten für Fragen des sexuellen Kindesmissbrauchs zusammengefasst [19]. Die Autor:innen schließen darin mit dem Fazit, dass für Deutschland aufgrund von nicht vergleichbaren Studiendesigns und Operationalisierungen sexueller Gewalt keine genauen Häufigkeitsangaben möglich seien [19]. Die Studienlage zur Häufigkeit sexueller Gewalterfahrungen im Jugend- und Erwachsenenalter ist für Deutschland deutlich schlechter als für solche Ereignisse im Kindesalter – insbesondere in Hinblick auf repräsentative Befragungen [14, 20–23]. International und national lassen sich auch hier deutlich mehr Studien zu Frauen finden, wobei Studien zu Gewalt ausgehend von sexuellen Intimpartnern im Vordergrund stehen [2, 7, 22, 24–26].

Aufgrund der dargestellten Forschungslücken verfolgt die vorliegende Studie im Rahmen der bundesweiten Survey-Untersuchung „Gesundheit und Sexualität in Deutschland“ (GeSiD) das Ziel, für Deutschland bevölkerungsrepräsentative Prävalenzdaten für 2 unterschiedliche Formen sexueller Gewalt^1^ im Kindesalter und über die Lebensspanne hinweg zu erheben: (versuchte/vollzogene) sexuelle Berührung und (versuchter/vollzogener) Sex gegen den eigenen Willen. Das gewählte Forschungsdesign orientiert sich dabei an dem dritten National Survey of Sexual Attitudes and Lifestyles (Natsal-3) aus dem Vereinigten Königreich, welcher 2013 erstmalig auch bevölkerungsrepräsentative Daten zur Prävalenz sexueller Gewalt und zu den Zusammenhängen mit Gesundheitsvariablen einschloss [7].

## Methode

### Datenerhebung

In der Studie „Gesundheit und Sexualität in Deutschland“ (GeSiD; [27–29]) wurden *N* = 4955 Frauen und Männer aus dem gesamten Bundesgebiet von Oktober 2018 bis September 2019 mittels computerassistierter persönlicher Interviews (Computer-assisted Personal Interview – CAPI) mit einem großen Selbstausfülleranteil (Computer-assisted Self Interview – CASI) befragt. Die Befragung erfolgte durch Interviewer:innen des sozialwissenschaftlichen Erhebungsinstituts KantarEmnid. Alle Fragen zu sexueller Gewalt wurden im Selbstausfülleranteil gestellt. Von allen Befragten lag eine schriftliche informierte Einwilligung vor. Die Teilnahmequote betrug 30,2 % (Response Rate 4 der American Association for Public Opinion Research; [30]). Die GeSiD-Daten wurden dahin gehend gewichtet, dass sie der deutschen Bevölkerung hinsichtlich des Alters, Geschlechts, der Bildung, Nationalität und geografischen Lage entsprechen. Eine ausführlichere Beschreibung des methodischen Vorgehens inklusive der Datenerhebung, der Teilnahmequote (mit besonderer Berücksichtigung der angewandten Berechnungsmethode), der Gewichtungsmethode und der Repräsentativität der Daten ist aufgrund des Umfangs in einer eigenständigen Publikation nachzulesen [31].

### Verwendete Items und Datenauswertung

#### Sexuelle Gewalt.

In der vorliegenden Studie wurden 2 Formen selbstberichteter sexueller Gewalt unterschieden: (versuchter/vollzogener) Sex gegen den eigenen Willen (Sex_gegen_Willen) und (versuchte/vollzogene) sexuelle Berührung gegen den eigenen Willen (Berührung_gegen_Willen). Sex_gegen_Willen wurde erfasst in Anlehnung an den britischen Natsal-3-Survey [7] mit der Frage: „Hat jemals eine Person gegen Ihren Willen oralen, analen oder vaginalen Sex (Geschlechtsverkehr) mit Ihnen gehabt oder dies versucht?“ (Antwortmöglichkeiten: 1 – Nein; 2 – Ja, ist passiert bzw. versucht worden; 3 – keine Angabe.) Berührung_gegen_Willen wurde erfasst in Anlehnung an die Studie von Krahé und Berger [32], das Item 20 des Childhood Trauma Questionnaire (CTQ; [33]) und das Item 3 des Adverse Childhood Experiences Questionnaire (ACE; [34]) mit der Frage: „Hat jemals eine Person gegen Ihren Willen versucht, Sie sexuell zu berühren oder Sie dazu zu bringen, ihn/sie zu berühren?“ (Antwortmöglichkeiten: 1 – Nein; 2 – Ja, ist passiert bzw. versucht worden; 3 – keine Angabe.) Das *Alter bei dem Ereignis* wurde erfragt mit: „Wie alt waren Sie, als dies das erste Mal geschah?“ [35]. Für die Berücksichtigung eines *Altersabstands von mindestens 5 Jahren zur/zum Beschuldigten* wurde bei Ereignissen vor dem 14. Lebensjahr zusätzlich folgende Frage ausgewertet: „War die Person damals mindestens 5 Jahre älter als Sie?“ (Antwortmöglichkeiten: 1 – Nein; 2 – Ja, die Person war (ungefähr) … Jahre alt; 3 – keine Angabe.) Die Kombination von Altersabstand von mindestens 5 Jahren zur/zum Beschuldigten und einem Ereignis in einem jüngeren Alter als 14 Jahre soll den Vergleich zu früheren Forschungsarbeiten ermöglichen, welche diese Definition für sexuellen Kindesmissbrauch gewählt haben [36].

#### Soziodemografische Faktoren.

Das Alter wurde in Jahren erfragt und in 6 *Altersgruppen* (18–25, 26–35, 36–45, 46–55, 56–65, 66–75 Jahre) klassifiziert. Die Einteilung in die dichotome Variable *Geschlecht (Mann/Frau)* entspricht den von den Einwohnermeldeämtern übermittelten Geschlechterangaben und steuerte die Auswahl der Fragen (Form für Männer oder Form für Frauen). Keines der Einwohnermeldeämter übermittelte den in Deutschland vor Kurzem eingeführten Geschlechtereintrag „divers“. Befragte hatten die Möglichkeit, zu Beginn des Interviews die Geschlechterangabe (und damit den verwendeten Fragebogen) zu verändern – hiervon machte allerdings niemand Gebrauch.^2^

Der *Deprivationsindex* zur Abbildung regionaler sozioökonomischer Unterschiede und sozialer Benachteiligung wurde erfasst nach dem „German Index of Socioeconomic Deprivation“ [37]. Der Index wird für die Nutzung in der Forschung und Gesundheitsberichterstattung des Bundes und der Länder bereitgestellt und soll dazu beitragen, neue Datenquellen für die Analyse des Zusammenhangs von sozialer Ungleichheit und Gesundheit zu erschließen. Er basiert auf den Merkmalen Beruf, Bildung und Einkommen und leistet einen Beitrag zur Erklärung regionaler Unterschiede in der Gesundheit. In der Aufschlüsselung nach Quintilen bedeutet das erste Quintil eine „niedrige Deprivation“ und das 5. Quintil die „höchste Deprivation“.

#### Gesundheitsbezogene Faktoren.

Die* Lebensqualität *wurde mit der Frage erhoben: „Wie zufrieden sind Sie gegenwärtig – alles in allem – mit Ihren Leben?“ Es standen 7 Antwortmöglichkeiten von (1) „überhaupt nicht zufrieden“ bis (7) „vollkommen zufrieden“, wobei für die Auswertung die beiden niedrigsten zu „niedrig“, 3–5 zu „mittel“ und die beiden höchsten zu „hoch“ zusammengefasst wurden.

*Allgemeiner Gesundheitszustand* wurde erfasst mit der Frage: „Wie ist Ihr Gesundheitszustand im Allgemeinen?“, mit den 5 Antwortmöglichkeiten „sehr gut“, „gut“, „mittelmäßig“, „schlecht“ und „sehr schlecht“, wobei die ersten und letzten beiden für die Auswertung zusammengefasst wurden.

*Chronische Erkrankungen und Behinderungen* wurden erfasst mit der Frage: „Leiden Sie unter einer chronischen Erkrankung oder Behinderung? Und wenn Ja: Beeinträchtigen diese Ihr Sexualleben?“, mit den Antwortmöglichkeiten „Nein“, „Ja, ich habe solche Erkrankungen, aber sie beeinträchtigen nicht mein Sexualleben“ und „Ja, ich habe solche Erkrankungen und sie haben Auswirkungen auf mein Sexualleben“.

*Depressionsbehandlung im letzten Jahr* und *Behandlung aufgrund einer anderen psychischen Erkrankung im letzten Jahr* wurde mit der Frage erfasst: „Sind Sie in den letzten 12 Monaten wegen einer der folgenden Erkrankungen behandelt (therapiert) worden?“ (Antwortmöglichkeit „Ja“: „Depression“ resp. „eine andere psychische Erkrankung“.)

Der *Body-Mass-Index* (BMI) wurde aus Körpergröße („Wie groß sind Sie?“) und Körpergewicht der Befragten („Und Ihr Gewicht in kg?“) berechnet. Die Körpergröße wurde mit einem Freitextfeld als Angabe in cm erfasst. Das Gewicht in kg wurde gruppiert in 5‑Kilogramm-Schritten erfasst. Für die Berechnung des BMI wurden jeweils die Gruppenmittelwerte gebildet (also aus 50–54 kg: 52 kg). Die beiden Extremgruppen „unter 50 kg“ und „130 kg und mehr“ wurden analog auf 47 kg beziehungsweise 132 kg gesetzt.

*Riskantes Trinkverhalten* wurde mit dem validierten Screener Audit‑C (Alcohol Use Disorders Identification Test) erfasst [38]. Als Risikokonsum wurde ein Wert über 3 für Männer beziehungsweise über 2 für Frauen gewertet. Der *Zigarettenkonsum* wurde erfasst mit der Frage: „Rauchen Sie gegenwärtig?“ (Ja/Nein).

Zusätzlich wurde *Drogenkonsum im letzten Jahr* erfasst mit: „Haben Sie in den letzten 12 Monaten eine der folgenden Substanzen zu sich genommen?“. Die Antwortmöglichkeiten wurden wie folgt geclustert: 1 – Nein; 2 – Ja, ausschließlich Cannabis („Ja, Cannabis (Marihuana, Gras, Haschisch)“); 3 – Ja, andere („Ja, Amylnitrit (Poppers)“; „Ja, etwas anderes“).

### Statistische Auswertung

Alle Auswertungen wurden mit dem Modul „Komplexe Stichprobe“ der Datenanalysesoftware IBM SPSS Statistics für Windows (Version 25) durchgeführt, das für Gewichtung, Clusterung und Stratifizierung der Daten adjustiert. Aufgrund der vorgenommenen Gewichtung der Daten wird auf eine ausführliche Stichprobenbeschreibung verzichtet und auf die Projektpublikation zum methodischen Vorgehen verwiesen [31].

Für die sexuellen Ereignisse vor dem 14. Lebensjahr wird die Lebenszeitprävalenz in Prozent berichtet, einschließlich der 95 %-Konfidenzintervalle (KI) in Tab. 1 zum einen ohne Berücksichtigung weiterer Variablen berichtet, zum anderen mit der Berücksichtigung eines Mindestaltersabstands von 5 Jahren zur/zum Beschuldigten. Die Lebenszeitprävalenz von Berührung_gegen_Willen in Prozent, einschließlich 95 %-KI, wird zum einen für alle Studienteilnehmenden dargestellt, zum anderen exklusive der Teilstichprobe, die zusätzlich auch Sex_gegen_Willen (Sex_g_W) berichtet (Berührung_gegen_Willen exkl. Sex_g_W). Differenziert für die ausgewählten soziodemografischen und gesundheitsbezogenen Variablen werden die Lebenszeitprävalenzen der abhängigen Variablen Sex_gegen_Willen und Berührung_gegen_Willen (exkl. Sex_g_W) für die Gesamtstichprobe (Tab. 2 und 3) bzw. der Gruppe mit Erlebnissen im Kindesalter bei zusätzlichem mind. fünfjährigen Altersabstand zur/zum Beschuldigten (Tab. 4 und 5) berichtet. Mittels logistischer Regression wurden Adjusted Odds Ratios (AOR) berechnet, um mögliche Zusammenhänge zu den demografischen und gesundheitsbezogenen Variablen zu untersuchen. Sämtliche Modelle wurden für Alter adjustiert und für Geschlecht stratifiziert (Tab. 2 und 3) bzw. adjustiert (Tab. 4 und 5). Im Onlinematerial findet sich darüber hinaus für alle Modelle die zusätzliche Adjustierung für Bildung und Deprivationsindex (Tabellen A1–A3).

**Table 1:** 

	Gesamtstichprobe		Frauen		Männer	
	% [KI]	*n* (ungewichtet; gewichtet)	% [KI]	*n* (ungewichtet; gewichtet)	% [KI]	*n* (ungewichtet; gewichtet)
**Ereignisse über die Lebensspanne**						
Sex_gegen_Willen	9,0 [8,1–10,0]	4891; 4881	14,9 [13,4–16,6]	2579; 2425	3,1 [2,4–4,1]	2312; 2456
Berührung_gegen_Willen	26,9 [25,5–28,3]	4882; 4866	40,8 [38,6–43,1]	2575; 2411	13,2 [11,7–14,8]	2307; 2455
Berührung_gegen_Willen (exkl. Sex_g_W)^a^	21,0 [19,7–22,4]	4423; 4431	32,2 [29,9–34,5]	2186; 2050	11,4 [10,1–12,9]	2237; 2381
**Ereignisse in der Kindheit**						
*Alter bei Erlebnis <* *14* *J.*						
Sex_gegen_Willen	2,1 [1,7–2,6]	4873; 4852	3,1 [2,4–4,0]	2565; 2402	1,2 [0,8–1,7]	2308; 2451
Berührung_gegen_Willen	7,5 [6,7–8,4]	4831; 4803	12,2 [10,8–13,7]	2534; 2363	2,9 [2,1–3,9]	2297; 2439
Berührung_gegen_Willen (exkl. Sex_g_W)^a^	5,0 [4,2–5,9]	4384; 4383	8,6 [7,2–10,2]	2155; 2014	2,0 [1,4–2,8]	2229; 2369
*Alter bei Erlebnis <* *14* *J. und Altersabstand zur/m Beschuldigten mind. 5* *J.*						
Sex_gegen_Willen	1,8 [1,4–2,3]	4860; 4838	2,8 [2,1–3,7]	2556; 2393	0,8 [0,5–1,3]	2304; 2445
Berührung_gegen_Willen	6,1 [5,4–6,9]	4774; 4745	10,4 [9,1–11,9]	2487; 2319	2,0 [1,4–2,8]	2287; 2426
Berührung_gegen_Willen (exkl. Sex_g_W)^a^	4,0 [3,3–4,8]	4339; 4338	7,1 [5,8–8,7]	2118; 1981	1,3 [0,0–2,0]	2221; 2357

*KI* 95 %-Konfidenzintervall, *n* Stichprobengröße

^a^Exkl. Sex_g_W: Teilstichprobe mit Erlebnis von Berührung_gegen_Willen, die nicht zusätzlich Sex_gegen_Willen berichtet hat

**Table 2:** 

	Frauen				Männer			
	Sex_gegen_Willen, % [95 % KI]	Age-adjusted Odds Ratios [KI]	*p*	*n* (ungewichtet; gewichtet)	Sex_gegen_Willen, % [95 % KI]	Age-adjusted Odds Ratios [KI]	*p*	*n* (ungewichtet; gewichtet)
**Gesamt**	14,9 [13,4–16,6]	–	–	2579; 2425	3,1 [2,4–4,1]	–	–	2312; 2456
**Altersgruppe**			0,021				0,062	
18- bis 25-Jährige	16,9 [13,1–21,5]	1,00		373; 279	3,0 [1,6–5,7]	1,00		385; 308
26- bis 35-Jährige	16,2 [13,2–19,7]	0,95 [0,64–1,41]		374; 419	3,8 [2,3–6,2]	1,28 [0,60–2,72]		535; 445
36- bis 45-Jährige	14,2 [10,5–18,9]	0,81 [0,53–1,24]		375; 396	5,6 [3,2–9,7]	1,92 [0,76–4,86]		375; 398
46- bis 55-Jährige	13,8 [10,3–18,3]	0,79 [0,50–1,24]		376; 520	2,9 [1,6–5,1]	0,96 [0,38–2,40]		360; 537
56- bis 65-Jährige	19,3 [15,2–24,1]	1,17 [0,79–1,75]		377; 467	1,9 [0,9–4,0]	0,63 [0,23–1,68]		373; 458
66- bis 75-Jährige	8,6 [5,4–13,5]	0,46 [0,26–0,83]		378; 346	1,3 [0,5–3,2]	0,41 [0,12–1,36]		284; 309
**Bildung**			0,450				0,798	
Niedrig	14,5 [11,6–18,1]	1,00		454; 689	2,5 [1,5–4,1]	1,00		537; 848
Mittel	13,7 [11,3–16,5]	0,89 [0,63–1,25]		877; 854	3,2 [1,8–5,6]	1,21 [0,56–2,62]		664; 708
Hoch	16,5 [14,3–18,9]	1,08 [0,77–1,52]		1248; 882	3,6 [2,4–5,4]	1,26 [0,65–2,44]		1111; 901
**Deprivationsindex**			0,412				0,648	
1 (niedrigstes Quintil)	15,7 [13,1–18,7]	1,00		850; 756	2,6 [1,6–4,3]	1,00		691; 763
2–4	15,1 [13,0–17,5]	0,95 [0,73–1,25]		1410; 1325	3,4 [2,4–4,9]	1,33 [0,72–2,48]		1274; 1330
5 (höchstes Quintil)	12,7 [9,5–16,8]	0,76 [0,51–1,15]		319; 344	3,1 [1,7–5,8]	1,26 [0,56–2,81]		347; 363
**Lebensqualität**			0,000				0,000	
Hoch	11,2 [9,3–13,3]	1,00		1441; 1358	2,1 [1,3–3,4]	1,00		1348; 1382
Mittel	19,2 [16,7–22,0]	1,86 [1,44–2,41]		1081; 1003	3,8 [2,7–5,1]	1,80 [0,98–3,30]		918; 1016
Niedrig	29,9 [17,4–46,2]	3,33 [1,59–6,97]		55; 60	16,8 [7,3–34,0]	10,00 [3,68–27,16]		40; 54
**Allgemeiner Gesundheitszustand**			0,000				0,000	
Sehr gut/gut	12,4 [10,8–14,3]	1,00		2001; 1811	2,9 [2,1–3,9]	1,00		1898; 1952
Mittelmäßig	20,8 [17,2–25,1]	2,07 [1,52–2,83]		474; 492	2,8 [1,5–5,2]	1,35 [0,67–2,75]		335; 403
Schlecht/sehr schlecht	31,5 [21,7–43,2]	3,82 [2,20–6,63]		94; 109	9,8 [5,1–18,0]	6,29 [2,71–14,62]		75; 97
**Chronische Erkrankung oder Behinderung**			0,000				0,026	
Nein	11,4 [9,8–13,2]	1,00 []		1769; 1625	2,7 [1,9–3,8]	1,00 []		1658; 1697
Ja	23,5 [19,7–27,6]	2,66 [1,95–3,63]		754; 738	3,9 [2,6–5,9]	1,95 [1,08–3,52]		612; 705
**Depressionsbehandlung im letzten Jahr**			0,000				0,014	–
Nein	12,6 [11,1–14,3]	1,00		2310; 2158	2,8 [2,1–3,8]	1,00		2189; 2317
Ja	33,9 [28,0–40,3]	3,48 [2,53–4,78]		269; 267	8,3 [4,0–16,5]	2,97 [1,25–7,06]		123; 139
**Behandlung aufgrund anderer psychischer Erkrankung im letzten Jahr**			0,000				0,000	
Nein	14,0 [12,4–15,7]	1,00		2454; 2316	2,8 [2,1–3,7]	1,00		2248; 2388
Ja	35,1 [26,2–45,1]	3,14 [2,04–4,84]		125; 109	14,4 [6,8–28,0]	5,16 [2,30–11,55]		64; 68
**Body-Mass-Index**			0,665				0,598	
Normal: 18,5–25 kg/m^2^	14,5 [12,3–17,0]	1,00		1358; 1188	2,7 [1,8–4,1]	1,00		932; 928
Untergewichtig: < 18,5 kg/m^2^	21,1 [12,7–32,9]	1,48 [0,83–2,65]		81; 63	6,6 [1,5–25,1]	2,50 [0,49–12,65]		23; 26
Übergewichtig: 25–30 kg/m^2^	15,2 [12,7–18,1]	1,10 [0,80–1,51]		668; 663	3,2 [2,1–4,8]	1,20 [0,65–2,24]		913; 980
Adipös: > 30 kg/m^2^	14,5 [11,2–18,6]	1,06 [0,72–1,57]		454; 495	3,5 [2,2–5,6]	1,43 [0,72–2,85]		423; 497
**Riskantes Trinkverhalten**			0,147				0,055	
Nein	14,0 [12,0–16,3]	1,00		1403; 1389	3,8 [2,7–5,3]	1,00		1107; 1211
Ja	16,9 [14,5–19,7]	1,21 [0,93–1,57]		1046; 899	2,3 [1,5–3,5]	0,59 [0,35–1,01]		1120; 1140
**Rauchen (gelegentlich oder regelmäßig)**			0,017				0,121	
Nein	13,5 [11,7–15,6]	1,00		1833; 1679	2,4 [1,7–3,4]	1,00		1500; 1544
Ja	18,3 [15,7–21,3]	1,36 [1,06–1,76]		739; 739	4,1 [2,8–5,9]	1,50 [0,90–2,49]		801;900
**Drogenkonsum im letzten Jahr**			0,000				0,685	
Nein	14,2 [12,6–15,9]	1,00		2327; 2232	2,9 [2,2–3,9]	1,00		1893; 2090
Ja, ausschließlich Cannabis	23,9 [18,2–30,7]	1,87 [1,29–2,71]		196; 147	4,4 [2,3–8,2]	1,38 [0,59–3,25]		300; 258
Ja, andere	42,6 [24,9–62,4]	4,34 [1,90–9,91]		31; 20	5,0 [1,5–15,2]	1,48 [0,40–5,53]		87; 76

*KI* 95 %-Konfidenzintervall, *n* Stichprobengröße

**Table 3:** 

	Frauen						Männer					
	Alle Frauen		Frauen, die nicht zusätzlich Sex_gegen_Willen berichtet haben				Alle Männer		Männer, die nicht zusätzlich Sex_gegen_Willen berichtet haben			
	Berührung_g_W, % [95 % KI]	*n* (ungewichtet; gewichtet)	Berührung_g_W^a^, % [95 % KI]	Age-adjusted Odds Ratios [KI]	*p*	*n* (ungewichtet; gewichtet)	Berührung_g_W, % [95 % KI]	*n* (ungewichtet; gewichtet)	Berührung_g_W^a^, % [95 % KI]	Age-adjusted Odds Ratios [KI]	*p*	*n* (ungewichtet; gewichtet)
**Gesamt**	40,8 [38,6–43,1]	2575; 2411	32,2 [29,9–34,5]	–	–	2186; 2050	13,2 [11,7–14,8]	2307; 2455	11,4 [10,1–12,9]	–	–	2237; 2381
**Altersgruppe**					0,154						0,424	–
18- bis 25-Jährige	39,6 [34,3–45,2]	373; 279	29,2 [23,7–35,3]	1,00		311; 232	13,7 [9,7–18,9]	384; 308	12,2 [8,7–16,9]	1,00		375; 299
26- bis 35-Jährige	42,0 [37,4–46,7]	553; 407	32,9 [28,5–37,7]	1,19 [0,86–1,65]		467; 340	12,4 [9,3–16,4]	534; 446	10,4 [7,7–14,1]	0,84 [0,51–1,38]		515; 429
36- bis 45-Jährige	36,3 [31,2–41,8]	429; 397	28,7 [23,6–34,4]	0,98 [0,65–1,46]		369; 341	15,3 [11,2–20,5]	374; 397	13,4 [9,6–18,5]	1,11 [0,63–1,96]		358; 375
46- bis 55-Jährige	45,4 [40,2–50,6]	493; 521	37,7 [32,6–43,1]	1,47 [1,00–2,16]		422; 450	14,2 [10,4–19,3]	360; 539	11,8 [8,4–16,4]	0,96 [0,58–1,61]		347; 524
56- bis 65-Jährige	43,7 [38,2–49,3]	489; 466	32,7 [27,2–38,7]	1,18 [0,79–1,76]		398; 376	13,7 [10,3–17,9]	371; 455	12,5 [9,2–16,8]	1,03 [0,61–1,73]		364; 448
66- bis 75-Jährige	34,9 [28,2–42,4]	238; 342	28,7 [22,0–36,6]	0,98 [0,62–1,55]		219; 312	8,3 [5,4–12,5]	284; 309	7,4 [4,7–11,5]	0,57 [0,30–1,08]		278; 305
**Bildung**					0,011						0,003	
Niedrig	36,0 [31,2–41,1]	451; 677	27,1 [22,4–32,4]	1,00		382; 578	12,6 [9,9–16,1]	534; 847	11,2 [8,5–14,6]	1,00		518; 826
Mittel	40,2 [36,6–43,8]	875; 851	32,3 [28,7–36,1]	1,26 [0,92–1,71]		750; 734	9,3 [7,3–11,9]	664; 709	7,6 [5,8–9,9]	0,63 [0,40–0,99]		645; 687
Hoch	45,2 [42,1–48,4]	1249; 883	36,0 [32,7–39,4]	1,57 [1,16–2,14]		1054; 738	16,6 [13,9–19,7]	1109; 899	14,7 [12,2–17,6]	1,34 [0,90–1,98]		1074; 868
**Deprivationsindex**					0,194						0,117	
1 (niedrigstes Quintil)	43,4 [39,5–47,4]	847;753	34,8 [30,6–39,3]	1,00		713; 635	12,8 [10,3–15,7]	694;767	11,6 [9,2–14,5]	1,00		678; 748
2–4	40,7 [37,5–43,8]	1413;1322	31,7 [28,9–34,6]	0,85 [0,66–1,08]		1198; 1122	14,2 [12,1–16,5]	1267;1326	12,3 [10,4–14,4]	1,07 [0,77–1,48]		1223; 1281
5 (höchstes Quintil)	35,9 [29,8–42,4]	315;337	28,3 [21,7–35,9]	0,72 [0,48–1,07]		275; 293	10,2 [6,8–15,1]	346;362	8,0 [5,6–11,4]	0,66 [0,41–1,05]		336; 351
**Lebensqualität**					0,001						0,395	
Hoch	35,4 [32,3–38,5]	1438; 1351	28,5 [25,8–31,5]	1,00		1273; 1199	11,4 [9,5–13,5]	1345; 1379	10,6 [8,9–12,6]	1,00		1318; 1351
Mittel	47,5 [44,2–50,9]	1081; 1000	37,4 [33,7–41,3]	1,50 [1,22–1,85]		871; 808	14,7 [12,2–17,6]	919; 1020	12,3 [9,9–15,3]	1,16 [0,83–1,63]		882; 983
Niedrig	52,9 [37,3–67,8]	56; 61	34,0 [20,1–51,4]	1,28 [0,62–2,65]		42; 43	30,6 [15,1–52,2]	38; 52	17,9 [7,0–38,5]	1,88 [0,65–5,48]		32; 43
**Allgemeiner Gesundheitszustand**					0,784						0,039	
Sehr gut/gut	39,0 [36,3–41,8]	1995; 1797	31,8 [29,2–34,6]	1,00		1734; 1572	12,2 [10,6–14,0]	1892; 1949	10,7 [9,2–12,4]	1,00		1842; 1894
Mittelmäßig	46,3 [41,3–51,3]	474; 490	34,0 [28,9–39,5]	1,11 [0,83–1,48]		372; 388	14,7 [10,7–20,0]	336; 403	13,0 [9,1–18,2]	1,35 [0,86–2,12]		325; 393
Schlecht/sehr schlecht	49,7 [38,3–61,2]	95; 110	32,7 [21,6–46,1]	1,06 [0,59–1,91]		70; 77	26,7 [17,9–37,8]	75; 100	20,7 [11,8–33,7]	2,47 [1,20–5,10]		66; 90
**Chronische Erkrankung oder Behinderung**					0,081						0,000	
Nein	37,2 [34,4–40,2]	1758; 1608	30,7 [27,8–33,7]	1,00		1540; 1423	10,9 [9,3–12,9]	1655; 1696	9,6 [8,0–11,4]	1,00		1613; 1652
Ja	48,8 [44,5–53,2]	762; 744	35,6 [30,9–40,6]	1,28 [0,97–1,70]		593; 571	18,7 [15,5–22,4]	613; 708	16,4 [13,4–20,0]	2,09 [1,50–2,91]		586; 680
**Depressionsbehandlung im letzten Jahr**					0,043						0,020	
Nein	38,7 [36,3–41,1]	2302; 2140	31,4 [29,0–33,8]	1,00		2009; 1868	12,4 [11,0–14,0]	2183; 2313	10,9 [9,6–12,5]	1,00		2123; 2251
Ja	57,6 [50,6–64,4]	273; 272	40,3 [32,4–48,8]	1,47 [1,01–2,14]		177; 182	25,0 [17,3–34,7]	124; 142	19,9 [12,6–30,0]	1,95 [1,12–3,43]		114; 130
**Behandlung aufgrund anderer psychischer Erkrankung im letzten Jahr**					0,107						0,110	
Nein	40,0 [37,7–42,4]	2449; 2302	31,8 [29,5–34,3]	1,00		2104; 1978	12,6 [11,1–14,2]	2242; 2384	11,2 [9,8–12,7]	1,00		2180; 2319
Ja	58,4 [48,4-67,8]	126; 110	41,3 [30,9–52,4]	1,49 [0,92–2,42]		82; 72	31,4 [20,3–45,3]	65; 71	20,5 [10,4–36,4]	1,95 [0,86–4,41]		57; 62
**Body-Mass-Index**					0,072						0,721	
Normal: 18,5–25 kg/m^2^	43,1 [39,9–46,3]	1353; 1180	35,1 [31,9–38,4]	1,00		1157; 1008	13,6 [11,4–16,2]	933; 933	12,2 [10,2–14,7]	1,00		908; 908
Untergewichtig: < 18,5 kg/m^2^	47,4 [35,2–59,9]	80; 63	36,9 [24,7–51,0]	1,10 [0,62–1,98]		63; 49	21,3 [9,8–40,2]	23; 26	15,8 [6,4–34,0]	1,28 [0,47–3,46]		21; 24
Übergewichtig: 25–30 kg/m^2^	37,1 [32,6–41,9]	668; 657	27,7 [23,2–32,7]	0,69 [0,52–0,93]		562; 556	12,3 [9,8–15,4]	909; 975	10,5 [8,3–13,4]	0,85 [0,59–1,21]		882; 944
Adipös: > 30 kg/m^2^	39,1 [33,7–44,9]	458; 498	30,4 [24,4–37,2]	0,80 [0,57–1,12]		393; 427	13,9 [10,7–18,1]	421; 497	11,9 [8,7–16,0]	0,95 [0,63–1,45]		406; 482
**Riskantes Trinkverhalten**					0,001						0,656	
Nein	37,2 [34,2–40,3]	1402; 1382	29,0 [25,9–32,3]	1,00		1202; 1188	12,9 [11,0–15,0]	1108; 1213	11,0 [9,2–13,0]	1,00		1068; 1168
Ja	46,9 [43,3–50,5]	1045; 897	37,5 [33,8–41,4]	1,48 [1,18–1,86]		874; 745	13,3 [11,0–16,1]	1117; 1139	11,9 [9,7–14,5]	1,08 [0,78–1,50]		1090; 1114
**Rauchen (gelegentlich oder regelmäßig)**					0,069						0,915	
Nein	38,3 [35,8–40,9]	1829; 1668	30,4 [27,8–33,3]	1,00		1583; 1442	12,4 [10,6–14,4]	1498; 1541	11,2 [9,4–13,2]	1,00		1458; 1504
Ja	46,6 [42,3–50,9]	739; 736	36,2 [31,7–40,9]	1,26 [0,98–1,61]		596; 601	14,2 [11,7–17,1]	798; 902	11,8 [9,4–14,6]	1,02 [0,73–1,43]		769; 867
**Drogenkonsum im letzten Jahr**					0,000	–					0,037	
Nein	39,5 [37,0–41,9]	2324;2222	31,2 [28,8–33,7]	1,00		2001; 1906	12,1 [10,5–13,9]	1887;2085	10,5 [9,0–12,2]	1,00		1833; 2026
Ja, ausschließlich Cannabis	60,4 [50,6–69,4]	196;147	49,7 [38,6–60,9]	2,37 [1,50–3,74]		145; 112	18,2 [13,7–23,7]	301;259	15,3 [11,3–20,4]	1,59 [1,00–2,54]		289; 247
Ja, andere	60,6 [41,4–77,0]	31;20	31,3 [14,6–55,0]	1,12 [0,40–3,17]		19; 11	21,1 [11,9–34,6]	87;76	19,6 [10,5–33,8]	2,18 [1,01–4,72]		83; 72

*Berührung_g_W* Berührung_gegen_Willen, *KI* 95 %-Konfidenzintervall, *n* Stichprobengröße

^a^Berührung_gegen_Willen exkl. Sex_g_W

**Table 4:** 

	Frauen		Männer		Gesamtstichprobe			
	Sex_g_Willen % [95 % KI]	*n* (ungewichtet; gewichtet)	Sex_g_Willen % [95 % KI]	*n* (ungewichtet; gewichtet)	Sex_g_Willen % [95 % KI]	Age-adjusted Odds Ratios [KI]	*p*-Wert	*n* (ungewichtet; gewichtet)
**Gesamt**	2,8 [2,1–3,7]	2556; 2393	0,8 [0,5–1,3]	2304; 2445	1,8 [1,4–2,3]	–	–	4860; 4838
**Altersgruppe**							0,246	
18- bis 25-Jährige	2,0 [0,9–4,2]	372; 278	0,8 [0,2–3,3]	385; 308	1,4 [0,7–2,7]	1,00		757; 586
26- bis 35-Jährige	2,0 [1,0–3,9]	553; 410	0,7 [0,2–1,9]	532; 442	1,3 [0,7–2,3]	0,95 [0,40–2,23]		1085; 853
36- bis 45-Jährige	2,0 [0,9–4,3]	422; 392	1,0 [0,4–2,8]	374; 397	1,5 [0,8–2,7]	1,07 [0,41–2,78]		796; 789
46- bis 55-Jährige	3,1 [1,8–5,0]	486; 509	0,8 [0,3–2,3]	356; 530	1,9 [1,2–3,0]	1,40 [0,59–3,33]		842; 1039
56- bis 65-Jährige	4,7 [3,1–7,3]	486; 461	1,0 [0,4–3,0]	373; 458	2,9 [1,9–4,4]	2,12 [0,91–4,92]		859; 919
66- bis 75-Jährige	2,5 [0,9–6,5]	237; 343	0,5 [0,1–2,6]	284; 309	1,5 [0,6–3,7]	1,08 [0,35–3,31]		521; 653
**Bildung**							0,510	
Niedrig	2,8 [1,6–4,9]	445; 672	1,0 [0,4–2,2]	535; 844	1,8 [1,2–2,8]	1,00		980; 1516
Mittel	2,5 [1,7–3,8]	873; 846	0,6 [0,2–1,5]	663; 705	1,7 [1,1–2,4]	0,80 [0,45–1,42]		1536; 1551
Hoch	3,1 [2,1–4,3]	1238; 875	0,8 [0,4–1,6]	1106; 897	1,9 [1,4–2,7]	1,12 [0,60–2,08]		2344; 1772
**Deprivationsindex**							0,862	
1 (niedrigstes Quintil)	2,6 [1,7–4,2]	846; 751	0,6 [0,2–1,7]	689; 760	1,6 [1,0–2,6]	1,00		1535; 1511
2–4	2,7 [1,9–4,0]	1393; 1302	1,1 [0,7–1,9]	1270; 1327	1,9 [1,4–2,6]	1,16 [0,67–2,02]		2663; 2629
5 (höchstes Quintil)	3,5 [1,8–6,6]	317; 340	0,2 [0,0–1,1]	345; 358	1,8 [0,9–3,4]	1,05 [0,46–2,34]		662; 698
**Lebensqualität**							0,005	
Hoch	2,1 [1,4–3,2]	1432; 1346	0,5 [0,2–1,2]	1347; 1379	1,3 [0,9–1,9]	1,00		2779; 2724
Mittel	3,7 [2,5–5,4]	1070; 988	0,8 [0,4–1,7]	911; 1008	2,2 [1,6–3,2]	1,72 [1,00–2,95]		1981; 1997
Niedrig	4,8 [1,4–15,4]	52; 55	8,1 [2,5–23,3]	40; 54	6,5 [2,8–14,1]	4,95 [1,85–13,25]		92; 109
**Allgemeiner Gesundheitszustand**							0,000	
Sehr gut/gut	1,8 [1,3–2,7]	1991; 1796	0,7 [0,4–1,2]	1892; 1944	1,2 [0,9–1,7]	1,00		3883; 3740
Mittelmäßig	5,5 [3,7–8,1]	464; 479	1,0 [0,4–3,0]	335; 403	3,4 [2,4–4,9]	2,57 [1,58–4,19]		799; 882
Schlecht/sehr schlecht	7,5 [3,0–17,5]	91; 105	2,8 [0,7–11,4]	73; 95	5,3 [2,5–11,0]	4,13 [1,78–9,58]		164; 199
**Chronische Erkrankung oder Behinderung**							0,003	
Nein	1,9 [1,2–2,9]	1761; 1616	0,9 [0,5–1,4]	1654; 1693	1,4 [1,0–1,9]	1,00		3415; 3308
Ja	5,0 [3,5–7,0]	740; 718	0,8 [0,3–2,0]	609; 702	2,9 [2,1–4,0]	2,03 [1,28–3,21]		1349; 1419
**Depressionsbehandlung im letzten Jahr**							0,000	
Nein	2,0 [1,4–2,9]	2298; 2138	0,8 [0,5–1,3]	2182; 2308	1,4 [1,1–1,8]	1,00		4480; 4445
Ja	9,5 [6,4–13,9]	258; 255	1,2 [0,2–8,0]	122; 138	6,6 [4,4–9,6]	4,05 [2,46–6,68]		380; 393
**Behandlung aufgrund anderer psychischer Erkrankung im letzten Jahr**							0,000	
Nein	2,5 [1,9–3,4]	2434; 2286	0,7 [0,4–1,2]	2240; 2377	1,6 [1,2–2,1]	1,00		4674; 4664
Ja	9,4 [5,3–16,2]	122; 106	3,9 [0,9–15,0]	64; 68	7,3 [4,3–11,9]	4,32 [2,34–7,99]		186; 175
**Body-Mass-Index**							0,360	
Normal: 18,5–25 kg/m^2^	2,0 [1,3–3,2]	1343; 1171	0,6 [0,3–1,5]	929; 926	1,4 [0,9–2,2]	1,00		2272; 2097
Untergewichtig: < 18,5 kg/m^2^	3,2 [0,8–12,9]	81; 63	3,8 [0,5–24,5]	23; 26	3,4 [1,0–10,9]	2,13 [0,59–7,63]		104; 89
Übergewichtig: 25–30 kg/m^2^	3,3 [2,2–5,0]	663; 654	0,8 [0,4–1,8]	910; 974	1,8 [1,3–2,7]	1,49 [0,78–2,84]		1573; 1628
Adipös: > 30 kg/m^2^	3,5 [2,1–6,0]	451; 488	1,1 [0,4–2,7]	421; 495	2,3 [1,5–3,6]	1,65 [0,81–3,34]		872; 984
**Riskantes Trinkverhalten**							0,602	
Nein	3,0 [2,1–4,3]	1394; 1374	0,9 [0,5–1,6]	1105; 1207	2,0 [1,5–2,8]	1,00		2499; 2580
Ja	2,5 [1,6–3,8]	1036; 885	0,8 [0,4–1,8]	1115; 1136	1,6 [1,1–2,3]	0,87 [0,52–1,46]		2151; 2022
**Rauchen (gelegentlich oder regelmäßig)**							0,536	
Nein	2,8 [2,0–3,9]	1819; 1657	0,7 [0,4–1,2]	1497; 1542	1,8 [1,3–2,4]	1,00		3316; 3199
Ja	2,8 [1,8–4,4]	730; 729	1,1 [0,6–2,2]	797; 894	1.9 [1,3–2,7]	1,15 [0,74–1,78]		1527; 1622
**Drogenkonsum im letzten Jahr**							0,035	
Nein	2,8 [2,1–3,8]	2308; 2203	0,7 [0,4–1,2]	1886; 2080	1,8 [1,4–2,3]	1,00		4194; 4283
Ja, ausschließlich Cannabis	1,7 [0,6–4,8]	193; 145	2,3 [1,1–5,0]	299; 257	2,1 [1,1–3,8]	1,85 [0,87–3,93]		492; 402
Ja, andere	11,4 [4,2–27,3]	30; 19	0,6 [0,1–4,5]	87; 76	2,8 [1,1–6,8]	3,44 [1,26–9,36]		117; 95

*Sex_g_Willen* Sex_gegen_Willen, *KI* 95 %-Konfidenzintervall, *n* Stichprobengröße

**Table 5:** 

	Frauen		Männer		Gesamtstichprobe			
	Berührung_g_W*; % [95 % KI]	*n* (ungewichtet; gewichtet)	Berührung_g_W*; % [95 % KI]	*n* (ungewichtet; gewichtet)	Berührung_g_W*; % [95 % KI]	Age-adjusted Odds Ratios [KI]	*p*	*n* (ungewichtet; gewichtet)
**Gesamt**	7,1 [5,8–8,7]	2118; 1981	1,3 [0,0–2,0]	2221; 2357	4,0 [3,3–4,8]	–	–	4339; 4338
**Altersgruppe**							0,005	
18- bis 25-Jährige	2,4 [1,1–5,2]	301; 226	0,7 [0,2–2,4]	372; 293	1,4 [0,7–2,8]	1,00		673; 520
26- bis 35-Jährige	7,0 [5,0–9,9]	451; 326	1,1 [0,5–2,5]	512; 426	3,7 [2,7–5,0]	2,65 [1,31–5,37]		963; 752
36- bis 45-Jährige	5,0 [3,1–7,9]	360; 327	1,3 [0,6–3,0]	355; 372	3,0 [2,0–4,6]	2,06 [0,90–4,70]		715; 698
46- bis 55-Jährige	9,6 [6,7–13,6]	407; 432	2,8 [1,4–5,5]	343; 517	5,9 [4,3–8,1]	4,28 [1,99–9,19]		750; 949
56- bis 65-Jährige	8,9 [6,0–13,1]	386; 365	1,2 [0,5–3,0]	362; 445	4,7 [3,2–6,7]	3,36 [1,45‑7,82]		748; 810
66- bis 75-Jährige	7,4 [4,4–12,3]	213; 305	0,1 [0,0–0,9]	277; 303	3,8 [2,2–6,4]	2,47 [1,04–5,89]		490; 608
**Bildung**							0,358	
Niedrig	6,6 [4,2–10,4]	367; 549	0,6 [0,2–2,2]	512; 815	3,0 [2,0–4,7]	1,00		879; 1364
Mittel	9,5 [7,4–12,3]	732; 718	0,4 [0,1–1,1]	642; 681	5,1 [3,9–6,6]	1,39 [0,84–2,29]		1374; 1399
Hoch	5,1 [3,7–7,0]	1019; 714	2,8 [1,8–4,3]	1067; 861	3,8 [3,0–4,9]	1,30 [0,77–2,21]		2086; 1574
**Deprivationsindex**							0,526	
1 (niedrigstes Quintil)	7,7 [5,2–11,2]	690; 614	1,6 [0,8–3,2]	673; 742	4,3 [3,0–6,2]	1,00		1363; 1356
2–4	6,9 [5,4–8,8]	1161; 1081	1,4 [0,8–2,4]	1213; 1266	4,0 [3,1–5,0]	0,85 [0,54–1,34]		2374; 2347
5 (höchstes Quintil)	6,7 [3,7–12,0]	267; 285	0,5 [0,2–1,4]	335; 349	3,3 [2,0–5,5]	0,70 [0,36–1,34]		602; 635
**Lebensqualität**							0,065	
Hoch	6,5 [4,9–8,6]	1237; 1166	1,1 [0,6–2,0]	1310; 1337	3,6 [2,8–4,7]	1,00		2547; 2503
Mittel	8,3 [6,4–10,6]	839; 772	1,8 [1,1–3,0]	875; 974	4,7 [3,7–5,9]	1,36 [0,95–1,95]		1714; 1746
Niedrig	3,3 [1,0–10,3]	42; 43	0	31; 42	1,7 [0,5–5,2]	0,41 [0,12–1,39]		73; 85
**Allgemeiner Gesundheitszustand**							0,363	
Sehr gut/gut	6,8 [5,3–8,6]	1681; 1518	1,2 [0,8–1,9]	1828; 1872	3,7 [2,9–4,6]	1,00		3509; 3390
Mittelmäßig	7,7 [5,0–11,8]	358; 374	2,0 [0,7–5,2]	324; 392	4,8 [3,3–6,9]	1,15 [0,70–1,87]		682; 766
Schlecht/sehr schlecht	12,7 [5,7–25,9]	69; 75	1,7 [0,4–7,0]	65; 89	6,7 [3,2–13,5]	1,77 [0,78–4,00]		134; 164
**Chronische Erkrankung oder Behinderung**							0,453	
Nein	6,8 [5,2–8,9]	1496; 1379	1,1 [0,7–1,8]	1603; 1636	3,7 [2,9–4,8]	1,00		3099; 3015
Ja	7,9 [5,7–10,8]	573; 554	1,9 [0,9–3,8]	580; 672	4,6 [3,4–6,1]	1,17 [0,77–1,79]		1153; 1226
**Depressionsbehandlung im letzten Jahr**							0,101	
Nein	6,9 [5,5–8,5]	1948; 1807	1,2 [0,8–1,9]	2108; 2228	3,8 [3,1–4,6]	1,00		4056; 4035
Ja	10,0 [6,0–16,1]	170; 174	3,2 [1,2–8,5]	113; 129	7,1 [4,5–11,1]	1,61 [0,91–2,84]		283; 303
**Behandlung aufgrund anderer psychischer Erkrankung im letzten Jahr**							0,039	
Nein	7,0 [5,7–8,6]	2037; 1910	1,2 [0,8–1,9]	2164; 2295	3,8 [3,2–4,7]	1,00		4201; 4205
Ja	9,8 [4,7–19,6]	81; 71	6,8 [2,4–17,5]	57; 62	8,4 [4,7–14,8]	2,10 [1,04–4,26]		138; 133
**Body-Mass-Index**							0,479	
Normal: 18,5–25 kg/m^2^	6,6 [4,9–8,8]	1119; 974	0,6 [0,3–1,2]	900; 896	3,7 [2,7–5,0]	1,00		2019; 1870
Untergewichtig: < 18,5 kg/m^2^	11,6 [4,6–26,6]	62; 48	0	21; 24	7,7 [3,0–18,4]	1,94 [0,68–5,50]		83; 72
Übergewichtig: 25–30 kg/m^2^	6,8 [4,4–10,5]	546; 540	1,7 [0,9–3,2]	878; 938	3,6 [2,5–5,2]	1,14 [0,69–1,87]		1424; 1478
Adipös: > 30 kg/m^2^	8,2 [5,5–12,1]	381; 410	2,2 [1,1–4,2]	402; 476	5,0 [3,5–7,1]	1,38 [0,83–2,30]		783; 885
**Riskantes Trinkverhalten**							0,065	
Nein	6,6 [5,2–8,4]	1170; 1155	0,9 [0,4–1,8]	1062; 1162	3,7 [3,0–4,7]	1,00		2232; 2317
Ja	7,8 [5,7–10,7]	844; 717	1,9 [1,2–3,2]	1082; 1100	4,3 [3,2–5,6]	1,41 [0,98–2,03]		1926; 1817
**Rauchen (gelegentlich oder regelmäßig)**							0,068	
Nein	6,1 [4,9–7,6]	1531; 1390	1,3 [0,8–2,3]	1447; 1487	3,7 [3,0–4,5]	1,00		2978; 2877
Ja	9,5 [6,8–13,1]	581; 585	1,4 [0,7–2,6]	764; 860	4,7 [3,4–6,3]	1,41 [0,97–2,05]		1345; 1446
**Drogenkonsum im letzten Jahr**							0,537	
Nein	7,3 [6,0–9,0]	1942; 1845	1,2 [0,7–1,9]	1823; 2009	4,1 [3,4–5,0]	1,00		3765; 3855
Ja, ausschließlich Cannabis	4,8 [1,9–11,5]	139; 107	2,7 [1,4–5,1]	285; 244	3,3 [1,9–5,8]	1,41 [0,70–2,81]		424; 350
Ja, andere	0	18; 11	2,7 [0,7–9,8]	81; 68	2,3 [0,6–8,5]	1,45 [0,34–6,21]		99; 79

Berührung_g_W* Teilstichprobe, die Berührung_gegen_Willen, aber nicht Sex_gegen_Willen berichtet hat, *KI* 95 %-Konfidenzintervall, *n* Stichprobengröße

## Ergebnisse

### Lebenszeitprävalenzen

Für Sex_gegen_Willen und Berührung_gegen_Willen über die Lebensspanne betrug die Lebenszeitprävalenz 9,0 % resp. 26,9 % (Tab. 1). Für solche Ereignisse vor dem 14. Lebensjahr waren es 2,1 % resp. 7,5 % und unter zusätzlicher Berücksichtigung eines oder einer um mindestens 5 Jahre älteren Beschuldigten: 1,8 % resp. 6,1 %. Frauen berichten wesentlich häufiger von solchen Ereignissen als Männer.

### Zusammenhang mit soziodemografischen und gesundheitsbezogenen Variablen

#### Sex_gegen_Willen über die Lebensspanne.

Die Prävalenz von Sex_gegen_Willen variierte in einem Großteil der untersuchten gesundheitsbezogenen Variablen bei Frauen und Männern, was sich in starken Zusammenhängen nach der Adjustierung für Alter (Age-adjusted Odds Ratios) zeigt (Tab. 2): Die höchsten Prävalenzraten fanden sich bei beiden Geschlechtern für Personen mit niedriger Lebensqualität, (sehr) schlechtem Gesundheitszustand, chronischer Erkrankung/Behinderung und Personen, die aufgrund einer Depression oder anderer psychischer Erkrankungen im letzten Jahr behandelt wurden. Für Frauen zeigte sich zusätzlich eine höhere Prävalenz bei denjenigen, die berichtet hatten, im letzten Jahr Nikotin oder psychogene Substanzen (ausschließlich Cannabis oder andere) konsumiert zu haben. Frauen der höchsten Altersgruppe (66–75 Jahre) berichteten am seltensten von Sex_gegen_Willen. Alle Zusammenhänge blieben für beide Geschlechter nach zusätzlicher Adjustierung für Bildung und Deprivationsindex bestehen (s. Onlinematerial Tabelle A2). Für Männer zeigte sich nach der zusätzlichen Adjustierung ein Zusammenhang mit riskantem Trinkverhalten dahin gehend, dass Männer, die Sex_gegen_Willen erlebt hatten, weniger häufig einen riskanten Alkoholkonsum berichten (s. Onlinematerial Tabelle A1).

#### Berührung_gegen_Willen über die Lebensspanne.

Für Frauen und Männer, die Berührung_gegen_Willen, aber nicht zusätzlich Sex_gegen_Willen berichteten, sind die Zusammenhänge mit den soziodemografischen und gesundheitsbezogenen Variablen in Tab. 3 dargestellt. Bei Frauen zeigten sich die höchsten Prävalenzraten bei einer mittleren Lebensqualität, Depressionsbehandlung im letzten Jahr, riskantem Trinkverhalten und Cannabiskonsum im letzten Jahr. Männer berichteten häufiger von Berührung_gegen_Willen bei schlechtem Gesundheitszustand, chronischer Erkrankung/Behinderung, Depressionsbehandlung im letzten Jahr und Konsum psychogener Substanzen (ausschließlich Cannabis oder andere). Bei beiden Geschlechtern ergaben sich Unterschiede in Abhängigkeit vom Bildungsniveau. Bei Frauen zeigte sich nach Adjustierung für Alter, Bildung und Deprivationsindex zusätzlich ein Zusammenhang für Rauchen (s. Onlinematerial Tabelle A2).

#### Ereignisse im Kindesalter.

Für die Teilstichprobe, die das erste Mal Sex_gegen_Willen unter 14 Jahren bei berücksichtigtem fünfjährigen Mindestaltersabstand zur/zum Beschuldigten berichteten, zeigten sich Zusammenhänge für alle gesundheitsbezogenen Faktoren außer dem Body-Mass-Index (BMI), Rauchen und riskantem Trinkverhalten, nicht jedoch für soziodemografische Variablen (Tab. 4, Onlinematerial Tabelle A3). Für Berührung_gegen_Willen (exkl. Sex_g_W) fanden sich Zusammenhänge für die Altersgruppe und einer Behandlung aufgrund einer anderen psychischen Erkrankung als Depression (Tab. 5), bei zusätzlicher Adjustierung für Bildung und Deprivationsindex zusätzlich für aktuellen Nikotinkonsum (Onlinematerial Tabelle A3).

## Diskussion

### Lebenszeitprävalenz

Die GeSiD-Studie ermöglicht anhand einer für Deutschland bevölkerungsrepräsentativen Stichprobe Aussagen zur Lebenszeitprävalenz zum Vorkommen von Erfahrungen sexueller Gewalt über die Lebensspanne. Für Deutschland liegen damit erstmalig detaillierte Häufigkeitsangaben für Frauen und Männer und differenziert für spezifische Gruppen mit unterschiedlichem Gesundheitsstatus und ausgewählten soziodemografischen Merkmalen vor.

Die Lebenszeitprävalenz zu Berührung_gegen_Willen verdeutlicht den großen Anteil von Personen in Deutschland, die eine Erfahrung im Laufe ihres Lebens machten, die sie als sexualisiert wahrnehmen und mindestens als Grenzverletzung erleben. Es ist bei diesen Selbstberichten von sexuellen Erlebnissen wie Berührung_gegen_Willen nicht sicher, welche Ereignisse gemäß dem deutschen Strafrecht sanktioniert würden. Dass 40,8 % der Frauen und damit bedeutend mehr Frauen als Männer (13,2 %) sexualisierte Kontakte gegen ihren Willen berichten, wird gesellschaftlich seit Langem und erneut verstärkt unter anderem seit der #aufschrei-Debatte im Jahr 2013 in Deutschland und insbesondere seit der im Jahr 2017 von den USA ausgehenden #Me-Too-Bewegung diskutiert [39, 40]. Die mediale Beachtung und Berichterstattung haben sich durch diese Diskurse in den letzten Jahren in Deutschland stark verändert und das ungleiche Geschlechterverhältnis bei den Opfern wird wiederholt im Zusammenhang mit den ungleichen gesellschaftlichen Machtverhältnissen von Frauen und Männern diskutiert [40]. Inwiefern dies zu einer steigenden Zahl von Anzeigen und Verurteilungen sexueller Gewaltstraftaten oder gar zu einer Abnahme von sexueller Gewalt führt, ist derzeit nicht abzusehen.

Insgesamt deutet ein Vergleich mit anderen repräsentativen Studien nicht darauf hin, dass durch die Mitberücksichtigung des Versuchs von sexuellen Handlungen gegen den eigenen Willen in der GeSiD-Studie die Prävalenz durch den Einschluss (vermeintlich) trivialer Ereignisse überschätzt wird: Die GeSiD-Lebenszeitprävalenz für Sex_gegen_Willen liegt für beide Geschlechter zwischen den Ergebnissen der britischen Survey-Studie Natsal‑3 für versuchten und vollzogenen Sex [7]. Die GeSiD-Prävalenzschätzung für Sex_gegen_Willen unter 14 Jahren entspricht mit 2,1 % (mit Berücksichtigung des Altersabstands zur/zum Beschuldigten: 1,8 %) nahezu dem Ergebnis der Studie von Häuser und Kolleg:innen für schweren bis extremen sexuellen Kindesmissbrauch ([41]; 1,9 %) sowie der Studie von Stadler und Kolleg:innen für Penetration (oral/anal/vaginal bzw. mit Finger/Zunge/Gegenstand) unter 14 Jahren ([36]; 1,9 %). Die deutschen Häufigkeitsangaben, welche bei einer weiter gefassten Definition sexuellen Kindesmissbrauchs bei einer Erfassung mit dem international weitverbreiteten Instrument Childhood Trauma Questionnaire (CTQ) ermittelt werden, variieren je nach Auswertungsmethode stark [41, 42]. Vergleicht man diese CTQ-Ergebnisse mit dem Ergebnis der GeSiD-Studie für Berührung_gegen_Willen im Kindesalter, zeigte sich jedoch auch hier, dass die Häufigkeiten durch den Einschluss des Versuchs sexueller Berührungen nicht zu einer wesentlich höheren Prävalenzrate geführt haben: Die GeSiD-Prävalenzen für Berührung_gegen_Willen unter 14 Jahren (inklusive Sex_gegen_Willen) liegen mit 7,5 % (mit Berücksichtigung des Altersabstands zur/zum Beschuldigten: 6,1 %) im vergleichbaren Bereich nach der Auswertungsmethode des CTQ für sexuellen Kindesmissbrauch von Iffland und Kolleg:innen ([42]; 6,2 %) und fallen verglichen mit den Prävalenzraten für (leichten bis extremen) sexuellen Kindesmissbrauch von Häuser und Kolleg:innen ([41]; 12,5 %) deutlich niedriger aus.

Die GeSiD-Daten können somit genutzt werden, um ein differenziertes Bild der Häufigkeitsangaben je nach Definition der *Hands-on*-Ereignisse^3^ zu erlangen, aber auch um das Vorkommen in Abhängigkeit von soziodemografischen oder gesundheitsbezogenen Faktoren einzuordnen. So können auch die Ergebnisse aus spezifischen deutschen Stichproben mit den repräsentativen GeSiD-Daten verglichen werden (wie z. B. sexuelle Kindesmissbrauchserfahrungen von über 60-Jährigen bei Glaesmer und Kolleg:innen [43]). Die GeSiD-Studie stellt damit insgesamt einen zusätzlichen, wesentlichen Baustein dar, sexuelle Gewalt/Grenzverletzung an Kindern und in der Gesamtpopulation Deutschlands zu beleuchten.

### Zusammenhänge von Sex_gegen_Willen mit gesundheitsbezogenen Faktoren

Bei Sex_gegen_Willen im Kindesalter ließen sich für alle erfassten gesundheitsbezogenen Variablen bedeutsame Zusammenhänge aufzeigen – mit Ausnahme von BMI, riskantem Trinkverhalten und Rauchen. Ein ähnliches Bild zeigte sich bei Berücksichtigung von solchen Ereignissen über die gesamte Lebensspanne für beide Geschlechter hinsichtlich einer bedeutsam höheren Prävalenz bei eingeschränkter Lebensqualität, schlechtem Gesundheitszustand, körperlicher Beeinträchtigung und dem Vorliegen psychischer Erkrankungen. Bei Ereignissen über die Lebensspanne zeigten sich höhere Prävalenzangaben zusätzlich für Frauen, die rauchen, und geringe Prävalenzangaben bei Männern, die einen riskanten Alkoholkonsum aufweisen. Für Sex_gegen_Willen im Kindesalter ließen sich keine Zusammenhänge mit soziodemografischen Ereignissen erkennen, bei Ereignissen über die Lebensspanne lediglich bei den Frauen dahin gehend, dass die älteste Alterskohorte am seltensten von Sex_gegen_Willen berichtet. Die Ergebnisse untermauern aber, dass sexuelle Gewalt ein Problem über alle gesellschaftlichen Gruppen hinweg darstellt. Aufgrund des verstärkten Auftretens bei psychisch und körperlich beeinträchtigten Personen sollte die Anamnese von sexuellen Grenzverletzungen unabhängig vom Geschlecht der Patient:innen ein fester Bestandteil der ärztlichen Praxis sein.

### Zusammenhänge von Berührung_gegen_Willen mit gesundheitsbezogenen Faktoren

Heterogener und weniger klar sind die Zusammenhänge mit der Gesundheit bei den im Vergleich zu Sex_gegen_Willen möglicherweise weniger schwerwiegenden sexuellen Grenzverletzungen in Form von versuchter/vollzogener sexueller Berührung. Bezüglich solcher Ereignisse im Kindesalter zeigte sich für Berührung_gegen_Willen ausschließlich ein deutlich höheres Vorkommen bei Personen, die wegen einer anderen psychischen Störung als Depression in den vergangenen 12 Monaten in Behandlung waren. Dies steht im Gegensatz zu den deutlichen Zusammenhängen von Sex_gegen_Willen mit einer Vielzahl der erfassten gesundheitsbezogenen Variablen. Bei Ereignissen über die Lebenspanne zeigten sich für Frauen und Männer Zusammenhänge mit einer Depressionsbehandlung und Drogenkonsum im letzten Jahr, ansonsten zeigten sich Unterschiede zwischen den Geschlechtern hinsichtlich der Zusammenhänge mit spezifischen gesundheitsbezogenen Variablen. Bei Frauen trat zusätzlich ein höheres Vorkommen bei der Gruppe mit einer eingeschränkten Lebensqualität und riskantem Trinkverhalten auf. Bei Männern scheint eine höhere Gefährdung bei einer chronischen Erkrankung und bei Vorliegen von weiteren psychischen Erkrankungen zu bestehen.

### Limitationen

Für diese Studie zur sexuellen Gewalt stand im GeSiD-Survey nur eine stark begrenzte Itemanzahl zur Verfügung. Der Fokus wurde deshalb auf 2 recht eng definierte Formen sog. Hands-on-Ereignisse sowie deren Versuch gelegt und umfasst nicht die gesamte Spannbreite sexueller Grenzverletzungen (u. a. exhibitionistische oder voyeuristische Handlungen, psychologische Gewaltformen). Bei den erfassten Ereignissen kann nicht zwischen versuchten und vollzogenen Ereignissen unterschieden werden. Da im Fokus dieser Studie der Zusammenhang mit gesundheitsbezogenen Faktoren stand, ist aus psychologischer und medizinscher Perspektive zu bemerken, dass der Versuch einer Vergewaltigung oder sexuellen Berührung keinesfalls ein triviales Ereignis für die berichtenden Personen darstellt. Bereits für weniger schwerwiegende Formen sexueller Belästigung – inklusive solcher in verbaler Form – konnten negative Auswirkungen auf die Gesundheit bei unterschiedlichen Altersgruppen nachgewiesen werden [45–48]. Die negativen Auswirkungen psychologischer Dimensionen von sexuellen Gewalterfahrungen auf die körperliche Gesundheit rücken zunehmend in den Blick [17]. Dazu zählt beispielsweise die Ausübung von Dominanz und Einschüchterung im Kontext der Erforschung gesundheitlicher Schädigungen infolge intimer Partnerschaftsgewalt [49].

Eine weitere Limitation der Studie stellt dar, dass die Anzahl untersuchbarer Teilnehmender bei bestimmten gesundheitsbezogenen Variablen sehr klein war, insbesondere die Gruppe mit Untergewicht, mit schlechtem allgemeinen Gesundheitszustand, mit niedriger Lebensqualität und Konsumenten von anderen Drogen als Cannabis [31]. Die Aussagekraft für diese Gruppen ist somit eingeschränkt und es bedarf des Vergleichs der Ergebnisse aus zukünftigen Studien, die diese Gruppen gezielt rekrutieren und untersuchen. Bei der Interpretation aller dargestellten Ergebnisse ist zudem zu beachten, dass die Zusammenhänge keine kausalen Schlüsse zulassen. Zukünftige Forschung sollte die zeitliche Reihenfolge von sexuellen Erlebnissen gegen den Willen und Erkrankungen/gesundheitsbezogenen Verhaltensweisen stärker in den Blick nehmen.

## Schlussfolgerung

Die GeSiD-Studie ermöglicht anhand einer bevölkerungsrepräsentativen Stichprobe Aussagen zur Lebenszeitprävalenz von selbstberichteter sexueller Gewalterfahrung im Kindesalter und über die Lebensspanne. Erstmalig stehen damit für Deutschland detaillierte Angaben zur Variabilität der Häufigkeiten in Abhängigkeit von bestimmten soziodemografischen Merkmalen und vom Gesundheitsstatus zur Verfügung. Die weltweiten Befunde zur deutlich höheren Prävalenz bei Frauen gegenüber Männern fanden sich auch in dieser Studie. Darüber hinaus machen die Ergebnisse deutlich, dass sexuelle Gewalt in Deutschland ein Problem über alle gesellschaftlichen Gruppen hinweg darstellt. Die Ergebnisse verdeutlichen zudem die ausgeprägten Zusammenhänge von (versuchtem/durchgeführtem) Sex_gegen_Willen und psychischer sowie somatischer Gesundheit – für Frauen und Männer. Besonders ausgeprägt sind die Zusammenhänge mit Gesundheit bei Sex_gegen_Willen im Kindesalter. Hinsichtlich Berührung_gegen_Willen im Kindesalter ist der Zusammenhang mit anderen psychischen Erkrankungen als Depression im Erwachsenenalter bedeutsam. Ärzt:innen sollten die gesundheitlichen Beeinträchtigungen infolge von sexueller Gewalt und sexualisierter Grenzverletzung im Blick haben, zusätzlich aber insbesondere für die erhöhte Gefährdung von Patient:innen mit chronischen Erkrankungen, Behinderungen oder psychischen Störungen sensibilisiert werden. In der ärztlichen und psychologischen Anamnese sollten Erlebnisse sexueller Gewalt unabhängig von Geschlecht, Alter und sozioökonomischem Status der Patient:innen routinemäßig exploriert werden. Wenngleich Männer weniger häufig sexuelle Ereignisse gegen den Willen berichten als Frauen, weist die GeSiD-Studie auf ebenfalls bedeutsame und teils ausgeprägtere Zusammenhänge mit gesundheitsbezogenen Variablen bei Männern hin.

## Supplementary Information





## Footnotes

1 Wir verwenden die Bezeichnungen „sexuelle Gewalt“ für selbstberichtete sexuelle Ereignisse gegen den eigenen Willen. Dabei handelt es sich um Berichte sexueller Ereignisse, bei denen aus – ggf. auch erst retrospektiver – Sicht der Studienteilnehmenden keine Einwilligung oder Einwilligungsfähigkeit vorlag.

2 Die verwendete Unterscheidung in Frauen und Männer berücksichtigt nicht die Tatsache, dass manche Befragte ihr Geschlecht als komplexer oder nichtbinär erleben. Die verwendete Variable wird in Einzelfällen dem individuellen Geschlechtserleben und dessen möglichen Veränderungen nicht gerecht.

3 „Hands-on“-Delikte werden im forensischen Kontext von „Hands-off“-Delikten abgegrenzt: Hands off bezeichnet Delikte ohne Berührung des Gegenübers (meist Exhibitionismus, Voyeurismus oder zwanghafter Konsum von Pornografie), Hands on umfasst Delikte mit direktem Körperkontakt [44].
