# Supplementary material for: Lifetime prevalences of nonconsensual sexual intercourse and touch and associations with health-related factors: Results from the German Health and Sexuality Survey (GeSiD)
Source: Bundesgesundheitsblatt Gesundheitsforschung Gesundheitsschutz. 2021 Oct 18;64(11):1339–54. [Article in German] doi: 10.1007/s00103-021-03434-6 (PMC8550730; doi:10.1007/s00103-021-03434-6)
Supplement: Supplementary file 1 [file 103_2021_3434_MOESM1_ESM.pdf]

Bundesgesundheitsblatt 11/2021

Onlinematerial zum Beitrag:

Lebenszeitprävalenz des Erlebens von Sex und sexueller Berührung gegen den eigenen Willen sowie Zusammenhänge mit gesundheitsbezogenen Faktoren. Ergebnisse aus der Studie Gesundheit und Sexualität (GeSiD) in Deutschland

Franziska Brunner[1], Safiye Tozdan[1], Verena Klein[1], Arne Dekker[1], Peer Briken[1]

[1] Institut für Sexualforschung, Sexualmedizin & Forensische Psychiatrie, Universitätsklinikum Hamburg-Eppendorf, Hamburg, Deutschland

Korrespondenzadresse
Dr. phil. Franziska Brunner
Institut für Sexualforschung, Sexualmedizin und Forensische Psychiatrie
Universitätsklinikum Hamburg-Eppendorf
Martinistraße 52
20246 Hamburg
Deutschland
f.brunner@uke.de

Inhalte:

Tabelle A1. Zusammenhänge von Sex_gegen_Willen mit soziodemografischen und gesundheitsbezogenen Faktoren

Tabelle A2. Zusammenhänge von Berührung_gegen_Willen (exkl. Sex_gegen_Willen) mit soziodemografischen und gesundheitsbezogenen Faktoren

Tabelle A3. Zusammenhänge für Sex_gegen_Willen und Berührung_gegen_Willen* im Kindesalter** mit soziodemografischen und gesundheitsbezogenen Faktoren

Tabelle A1. Zusammenhänge von Sex_gegen_Willen mit soziodemografischen und gesundheitsbezogenen Faktoren

	Frauen			Männer		
	Adjusted Odds Ratios* [95% KI]	p	n (ungewichtet; gewichtet)	Adjusted Odds Ratios* [95% KI]	p	n (ungewichtet; gewichtet)
**Altersgruppe**		.021			.074	
18- bis 25-Jährige	1.00		373; 279	1.00		385; 308
26- bis 35-Jährige	0.97 [0.65-1.44]		558; 416	1.25 [0.59-2.66]		535; 445
36- bis 45-Jährige	0.83 [0.54-1.28]		427; 396	1.90 [0.77-4.69]		375; 398
46- bis 55-Jährige	0.83 [0.52-1.31]		493; 520	0.96 [0.39-2.37]		360; 537
56- bis 65-Jährige	1.24 [0.82-1.89]		490; 467	0.64 [0.23-1.74]		373; 458
66- bis 75-Jährige	0.48 [0.26-0.90]		238; 346	0.42 [0.13-1.40]		284; 309
**Bildung**		.531			.079	
niedrig	1.00		454; 689	1.00		537; 848
mittel	0.89 [0.63-1.26]		877; 854	1.20 [0.54-2.65]		664; 708
hoch	1.07 [0.76-1.49]		1248; 882	1.28 [0.66-2.48]		1111; 901
**Deprivationsindex**		.486			.638	
1 (niedrigstes Quintil)	1.00		850; 756	1.00		691; 763
2-4	0.96 [0.73-1.25]		1410; 1325	1.34 [0.72-2.50]		1274; 1330
5 (höchstes Quintil)	0.78 [0.52-1.18]		319; 344	1.25 [0.55-2.85]		347; 363
**Lebensqualität**		.000			.000	
hoch	1.00		1441;1358	1.00		1348; 1382
mittel	1.87 [1.44-2.43]		1081; 1003	1.78 [0.96-3.30]		918; 1016
niedrig	3.39 [1.60-7.19]		55; 60	10.74 [3.75-30.70]		40; 54
**allgemeiner Gesundheitszustand**		.000			.000	
Sehr gut/gut	1.00		2001; 1811	1.00		1898; 1952
Mittelmäßig	2.12 [1.55-2.89]		474; 492	1.37 [0.65-2.89]		335; 403
Schlecht/sehr schlecht	3.93 [2.27-6.79]		94; 109	6.86 [2.74-17.21]		75; 97
**chronische Erkrankung oder Behinderung**		.000			.025	
nein	1.00		1769; 1625	1.00		1658; 1697
ja	2.66 [1.95-3.63]		754; 738	1.97 [1.09-3.55]		612; 705
**Depressionsbehandlung im letzten Jahr**		.000			.013	
nein	1.00		2310; 2158	1.00		2189; 2317
ja	3.47 [2.51-4.80]		269; 267	3.02 [1.27-7.20]		123; 139

**Behandlung aufgrund anderer psychischer Erkrankung im letzten Jahr**		.000			.000	
nein	1.00		2454; 2316	1.00		2248; 2388
ja	3.15 [2.04-4.86]		125, 109	5.06 [2.19-11.69]		64; 68
**Body-Mass-Index**		.656			.541	
normal: 18.5-25 kg/m^2	1.00		1358; 1188	1.00		932; 928
untergewichtig: <18.5 kg/m^2	1.46 [0.81-2.65]		81; 63	2.68 [0.56-12.84]		23; 26
übergewichtig: 25-30 kg/m^2	1.11 [0.81-1.53]		668; 663	1.20 [0.65-2.20]		913; 980
adipös: >30 kg/m^2	1.09 [0.74-1.61]		454; 495	1.46 [0.72-2.96]		432; 497
**riskantes Trinkverhalten**		.185			.039	
nein	1.00		1403; 1389	1.00		1107; 1211
Ja	1.20 [0.92-1.56]		1046; 899	0.57 [0.33-0.97]		1120; 1140
**Rauchen (gelegentlich oder regelmäßig)**		.009			.072	
nein	1.00		1833; 1679	1.00		1500; 1544
Ja	1.42 [1.09-1.83]		739; 739	1.61 [0.96-2.70]		801; 900
**Drogenkonsum im letzten Jahr**		.000			.674	
nein	1.00		2327; 2232	1.00		1893; 2090
ja, ausschließlich Cannabis	1.84 [1.27-2.68]		196; 147	1.38 [0.60-3.17]		300; 258
ja, andere	4.31 [1.88-9.88]		31; 20	1.49 [0.40-5.52]		87; 76

*adjustiert für Alter, Bildung und Deprivationsindex

Tabelle A2. Zusammenhänge von Berührung_gegen_Willen (exkl. Sex_gegen_Willen) mit soziodemografischen und gesundheitsbezogenen Faktoren

	Frauen			Männer		
	Adjusted Odds Ratios** [95% KI]	p	n (ungewichtet; gewichtet)	Adjusted Odds Ratios** [95% KI]	p	n (ungewichtet; gewichtet)
**Altersgruppe**		.094			.388	
18- bis 25-Jährige	1.00		311; 232	1.00		375; 299
26- bis 35-Jährige	1.19 [0.86-1.64]		467; 340	0.82 [0.49-1.35]		515; 429
36- bis 45-Jährige	1.00 [0.66-1.50]		369; 341	1.12 [0.63-1.98]		358; 375
46- bis 55-Jährige	1.60 [1.08-2.38]		422; 450	1.04 [0.61-1.77]		347; 524
56- bis 65-Jährige	1.33 [0.88-2.02]		398; 376	1.11 [0.65-1.89]		364; 448
66- bis 75-Jährige	1.15 [0.72-1.83]		219; 312	0.60 [0.31-1.14]		278; 305
**Bildung**		.015			.006	
niedrig	1.00		382; 578	1.00		518; 826
mittel	1.28 [0.93-1.75]		750; 734	0.67 [0.42-1.05]		645; 687
hoch	1.56 [1.15-2.12]		1054; 738	1.35 [0.91-2.01]		1074; 868
**Deprivationsindex**		.238			.288	
1 (niedrigstes Quintil)	1.00		713; 635	1.00		678; 748
2-4	0.85 [0.67-1.08]		1198; 1122	1.12 [0.80-1.55]		1223; 1281
5 (höchstes Quintil)	0.73 [0.49-1.10]		275; 293	0.78 [0.49-1.23]		336; 351
**Lebensqualität**		.001			.214	
hoch	1.00		1273; 1199	1.00		1318; 1351
mittel	1.53 [1.24-1.89]		871; 808	1.19 [0.85-1.68]		882; 983
niedrig	1.37 [0.66-2.82]		42; 43	2.36 [0.81-6.90]		32; 43
**allgemeiner Gesundheitszustand**		.623			.011	
Sehr gut/gut	1.00		1734; 1572	1.00		1842; 1894
Mittelmäßig	1.15 [0.86-1.55]		372; 388	1.47 [0.93-2.33]		325; 393
Schlecht/sehr schlecht	1.09 [0.59-2.01]		70; 77	2.95 [1.40-6.22]		66; 90
**chronische Erkrankung oder Behinderung**		.082			.000	
nein	1.00		1540;1423	1.00		1613;1652
ja	1.28 [0.97-1.69]		593; 571	2.11 [1.51-2.94]		586; 680
**Depressionsbehandlung im letzten Jahr**		.037			.018	
nein	1.00		2009; 1867	1.00		2123; 2251
ja	1.50 [1.03-2.18]		177; 182	1.96 [1.12-3.41]		114; 130
**Behandlung aufgrund anderer psychischer Erkrankung im letzten Jahr**		.103			.079	
nein	1.00		2104; 1978	1.00		2180; 2319
ja	1.50 [0.92-2.45]		82; 72	2.06 [0.92-4.62]		57; 62

**Body-Mass-Index**		.176			.714	
normal: 18.5-25 kg/m^2	1.00		1157; 1008	1.00		908; 908
untergewichtig: <18.5 kg/m^2	1.06 [0.59- 1.89]		63; 49	1.24 [0.46-3.33]		21; 24
übergewichtig: 25-30 kg/m^2	0.72 [0.54-0.97]		562; 556	0.87 [0.60-1.25]		882; 944
adipös: >30 kg/m^2	0.87 [0.61-1.24]		393; 427	1.05 [0.69-1.61]		406; 482
**riskantes Trinkverhalten**		.003			.652	
nein	1.00		1202; 1188	1.00		1068; 1168
ja	1.42 [1.13-1.78]		874; 745	1.08 [0.77-1.51]		1090; 1114
**Rauchen (gelegentlich oder regelmäßig)**		.016			.524	
nein	1.00		1583; 1442	1.00		1458; 1504
ja	1.36 [1.06-1.76]		596; 601	1.12 [0.79-1.59]		769; 867
**Drogenkosum im letzten Jahr**		.001			.042	
nein	1.00		2001; 1906	1.00		1833; 2026
ja, ausschließlich Cannabis	2.26 [1.42-3.60]		145; 112	1.55 [0.97-2.47]		289; 247
ja, andere	1.01 [0.35-2.89]		19; 11	2.20 [1.01-4.78]		83; 72

* Berührung_gegen_Willen (exkl. Sex_gegen_Willen); ** adujustiert für Alter, Bildung und Deprivations-Index

Tabelle A3. Zusammenhänge für Sex_gegen_Willen und Berührung_gegen_Willen* im Kindesalter** mit soziodemografischen und gesundheitsbezogenen Faktoren

	Sex_gegen_Willen			Berührung_gegen_Willen*		
	Adjusted Odds Ratios*** [95% KI]	p	n (ungewichtet; gewichtet)	Adjusted Odds Ratios*** [95% KI]	p	n (ungewichtet; gewichtet)
**Altersgruppe**		.258			.006	
18- bis 25-Jährige	1.00		757; 586	1.00		673; 520
26- bis 35-Jährige	0.96 [0.41-2.24]		1085; 853	2.62 [1.29-5.31]		963; 752
36- bis 45-Jährige	1.11 [0.42-2.91]		796; 789	1.99 [0.88-4.55]		715; 698
46- bis 55-Jährige	1.48 [0.61-3.61]		842; 1039	4.27 [1.94-9.37]		750; 949
56- bis 65-Jährige	2.25 [0.91-5.58]		859; 919	3.49 [1.47-8.30]		748; 810
66- bis 75-Jährige	1.14 [0.34-3.77]		521; 653	2.64 [1.08-6.42]		490; 608
**Bildung**		.504			.327	
niedrig	1.00		980; 1516	1.00		879; 1364
mittel	0.80 [0.45-1.43]		1536; 1551	1.43 [0.85-2.41]		1374; 1399
hoch	1.13 [0.61-2.09]		2344; 1772	1.29 [0.76-2.19]		2086; 1574
**Deprivationsindex**		.850			.489	
1 (niedrigstes Quintil)	1.00		1535; 1511	1.00		1363; 1356
2-4	1.17 [0.68-2.03]		2663; 2629	0.85 [0.54-1.34]		2374; 2347
5 (höchstes Quintil)	1.12 [0.49-2.56]		662; 698	0.67 [0.34-1.32]		602; 635
**Lebensqualität**		.005			.068	
hoch	1.00		2779; 2724	1.00		2547; 2503
mittel	1.73 [1.00-2.98]		1981; 1997	1.37 [0.96-1.96]		1714; 1746
niedrig	4.98 [1.82-13.63]		92; 109	0.43 [0.13-1.48]		73; 85
**allgemeiner Gesundheitszustand**		.000			.307	
Sehr gut/gut	1.00		3883; 3740	1.00		3509; 3390
Mittelmäßig	2.62 [1.58-4.37]		799; 882	1.19 [0.72-1.96]		682; 766
Schlecht/sehr schlecht	4.27 [1.88-9.70]		164; 199	1.85 [0.81-4.22]		134; 164
**chronische Erkrankung oder Behinderung**		.003			.446	
nein	1.00		3415; 3308	1.00		3099; 3015
ja	2.02 [1.28-3.21]		1349; 1419	1.18 [0.77-1.80]		1153; 1226
**Depressionsbehandlung im letzten Jahr**		.000			.100	
nein	1.00		4480; 4445	1.00		4056; 4035
ja	4.05 [2.43-6.75]		380; 393	1.61 [0.91-2.84]		283; 303
**Behandlung aufgrund anderer psychischer Erkrankung im letzten Jahr**		.000			.048	
nein	1.00		4674; 4664	1.00 []		4201; 4205
ja	4.41 [2.38-8.19]		186; 175	2.05 [1.01-4.19]		138; 133

**Body-Mass-Index**		.308			.383	
normal: 18.5-25 kg/m²	1.00		2272; 2097	1.00		2019; 1870
untergewichtig: <18.5 kg/m²	2.03 [0.55-7.51]		104; 89	1.93 [0.67-5.57]		83; 72
übergewichtig: 25-30 kg/m²	1.52 [0.80-2.89]		1573; 1628	1.18 [0.72-1.95]		1424; 1478
adipös: >30 kg/m²	1.73 [0.86-3.49]		872; 984	1.49 [0.89-2.51]		783; 885
**riskantes Trinkverhalten**		.572			.084	
nein	1.00		2499; 2580	1.00		2232; 2317
ja	0.86 [0.52-1.44]		2151; 2022	1.37 [0.96-1.97]		1926; 1817
**Rauchen (gelegentlich oder regelmäßig)**		.455			.036	
nein	1.00		3316; 3199	1.00		2978; 2877
ja	1.18 [0.76-1.85]		1527; 1622	1.49 [1.03-2.16]		1345; 1446
**Drogenkonsum im letzten Jahr**		.029			.596	
nein	1.00		4194; 4283	1.00		3765; 3855
ja, ausschließlich Cannabis	1.81 [0.89-3.71]		492; 402	1.37 [0.68-2.77]		424; 350
ja, andere	3.45 [1.27-9.37]		117; 95	1.39 [0.32-5.98]		99; 79

* Berührung_gegen_Willen (exkl. Sex_gegen_Willen); ** < 14 Jahren und Altersabstand zur/m Beschuldigten von mind. 5 Jahren; *** adjustiert für Alter, Bildung und Deprivationsindex
